# B-Cell Maturation Antigen (BCMA) as a Biomarker and Potential Treatment Target in Systemic Lupus Erythematosus

**DOI:** 10.3390/ijms251910845

**Published:** 2024-10-09

**Authors:** Jonas Martin, Qingyu Cheng, Sarah A. Laurent, Franziska S. Thaler, Anne Elisabeth Beenken, Edgar Meinl, Gerhard Krönke, Falk Hiepe, Tobias Alexander

**Affiliations:** 1 Department of Rheumatology and Clinical Immunology, Charité—Universitätsmedizin Berlin, 10117 Berlin, Germany; jonas.martin@charite.de (J.M.); cheng@drfz.de (Q.C.); anne.beenken@charite.de (A.E.B.); gerhard.kroenke@charite.de (G.K.); falk.hiepe@charite.de (F.H.); 2 Deutsches Rheuma-Forschungszentrum Berlin, a Leibniz Institute, 10117 Berlin, Germany; 3 Institute of Clinical Neuroimmunology, University Hospital, Ludwig-Maximilians-Universität München, 80336 Munich, Germanyfranziska.thaler@med.uni-muenchen.de (F.S.T.); edgar.meinl@med.uni-muenchen.de (E.M.); 4 Biomedical Center (BMC), Medical Faculty, Ludwig-Maximilians-Universität München, 82152 Martinsried, Germany

**Keywords:** SLE, BAFF, BCMA, TACI, lupus, B cells, plasma cells

## Abstract

The BAFF-APRIL system is crucial for the pathogenesis of systemic lupus erythematosus (SLE) by promoting B cell survival, differentiation and the maintenance of humoral autoimmunity. Here, we investigated the relationship of BCMA expression on B cell subsets with its ligands BAFF and APRIL, together with soluble BCMA, and with clinical and serologic variables in a cohort of 100 SLE patients (86 under conventional and 14 under belimumab therapy) and 30 healthy controls (HCs) using multicolor flow cytometry and ELISA. We found that BCMA expression in SLE patients was significantly increased on all B cell subsets compared to HCs, with all examined components of the BAFF-APRIL system being upregulated. BCMA expression was significantly increased on switched and unswitched memory B cells compared to naïve B cells, both in HCs and SLE. BCMA expression on B cells correlated with plasmablast frequencies, serum anti-dsDNA antibodies and complement consumption, while soluble BCMA correlated with plasmablast frequency, highlighting its potential as a clinical biomarker. Belimumab treatment significantly reduced BCMA expression on most B cell subsets and soluble TACI and contributed to the inhibition of almost the entire BAFF-APRIL system and restoration of B cell homeostasis. These results provide insights into the complex dysregulation of the BAFF-APRIL system in SLE and highlight the therapeutic potential of targeting its components, particularly BCMA, in addition to its use as a biomarker for disease activity.

## 1. Introduction

Systemic lupus erythematosus (SLE) is a prototypic systemic autoimmune disease characterized by multiple organ manifestations and autoantibody production against a variety of nuclear antigens that strongly contribute to disease pathogenesis [1]. Although the etiology of SLE has not yet been fully clarified, antibody-secreting cells, such as plasmablasts, as well as long-lived plasma cells and their precursor B cells, are crucial both for the development and perturbation of the disease [2,3,4]. Given their role in persistent autoantibody production, long-lived plasma cells represent novel cellular targets for biologic therapies in SLE, such as proteasome inhibitors [5] and anti-CD38 monoclonal antibodies [6,7]. Since plasma cells express high levels of BCMA, it is an attractive target to deplete autoreactive plasma cells. In fact, recent studies demonstrated the clinical efficacy of a dual CD19-BCMA-directed chimeric antigen receptor (CAR)-T cell therapy in SLE [8]. In addition, we recently utilized the CD3-BCMA bispecific antibody teclistamab, approved for multiple myeloma [9], in a refractory case of SLE, which resulted in a complete clinical and serologic remission despite the discontinuation of immunosuppressive therapies. The beneficial responses were associated with the depletion of plasma cells in the bone marrow and a preferential eradication of CD27^+^ memory B cells [10].

Among pathogenic B cell subsets, activated naïve [11,12,13,14] as well as double-negative memory B cells, particularly T-bet^+^CD11c^+^ age-associated B cells [15,16], CD27^−^ IgD^−^ CXCR5^−^ CD11c^+^ double negative [17,18,19,20] and CXCR5^−^ CD19^low^ plasmablast precursors [21], are newly identified subsets of autoreactive B cells that are expanded during flares of the disease and are implicated in humoral dysregulation of SLE. Although genetic predispositions and environmental factors have been identified in the pathogenesis of SLE, the generation and underlying molecular mechanisms of autoreactive B cells largely remain unclear. Important factors that activate B cells and stimulate them to differentiate into plasmablasts and plasma cells are soluble factors BAFF (B cell activating factor) and APRIL (a proliferation-inducing ligand). Both are ligands to the surface receptors B cell maturation antigen (BCMA) and transmembrane activator and CAML interactor (TACI) on B cells, and binding to them induces the proliferation and survival of B cells [22,23,24,25,26,27,28]. BAFF and APRIL have also been reported to protect plasma cells [29] and multiple myeloma cells [30] from apoptosis.

BCMA, a member of the tumor necrosis factor (TNF) receptor superfamily, is a membrane-bound surface receptor that is expressed on B cell lineage-derived cells, particularly on plasmablasts and plasma cells, along with a subset of activated CD38^+^ memory B cells [29]. The amount of its expression represents the state of B cell activation both in healthy individuals and SLE patients [26,31]. BCMA expression can be induced by stimulation with cytokines and is essential for the survival of long-lived plasma cells in the bone marrow [32]. BCMA is a short-lived protein and its expression is tightly regulated by multiple mechanisms. BCMA is ubiquitylated and degraded by the ubiquitin proteasome system in multiple myeloma [33]. Furthermore, BCMA is shed by gamma-secretase and then released into the blood, where it can be measured as soluble BCMA (sBCMA) by ELISA [34,35]. We previously reported elevated serum levels of sBCMA in a cohort of untreated SLE patients compared to healthy controls, with a strong correlation to disease activity [34]. These findings were confirmed in a cohort of SLE patients under treatment, suggesting the role of sBCMA as a biomarker for disease activity in SLE [36].

Previous studies investigating BCMA expression on immune cell subsets by flow cytometry demonstrated significantly increased BCMA expression on SLE B cells compared to healthy controls [26,37,38]. They also revealed correlations of BCMA expression on B cells with disease severity in SLE, yet with contradictory results. While one study reported increased BCMA expression on almost all B cell subsets and a positive correlation of BCMA expression on total B cells with disease activity [26], another study described decreased BCMA expression on almost all B cell subsets in a small cohort of SLE patients compared to healthy controls and a negative correlation between BCMA-expressing B cells and disease activity [39].

Based on the apparent role of BCMA in SLE pathogenesis, we aimed to analyze the expression of BCMA on freshly isolated B cell subsets in SLE patients under conventional and BAFF-targeting therapy with belimumab, which confirms and extends previous results, and demonstrate that increased BCMA expression on B cells is a common and reproducible feature of SLE. BCMA expression on B cells strongly correlates with the frequency of circulating plasmablasts and serum dsDNA antibody titers and is downregulated under belimumab treatment. We also observed higher BCMA expression levels on memory B cell subsets compared to naïve B cells, identifying BCMA as a potential therapeutic target for depleting plasma cells and their memory B cell precursors with monoclonal or bispecific antibodies, as well as BCMA-directed CAR-T cell therapies.

## 2. Results

### 2.1. B Cell Subset Distribution and Their BCMA Expression

To investigate the BCMA expression on peripheral blood B cell subsets and plasmablasts, we performed multicolor flow cytometry on freshly obtained peripheral blood after red blood cell lysis in a cohort of 100 SLE patients compared to 30 healthy controls (HCs). Patient demographics and disease characteristics are shown in Table 1. Patients were categorized according to their treatment into those receiving standard of care (SLE-SOC), defined as patients without biologics (*n* = 86), and those with belimumab treatment (SLE-BEL, *n* = 14).

**Figure 1:**
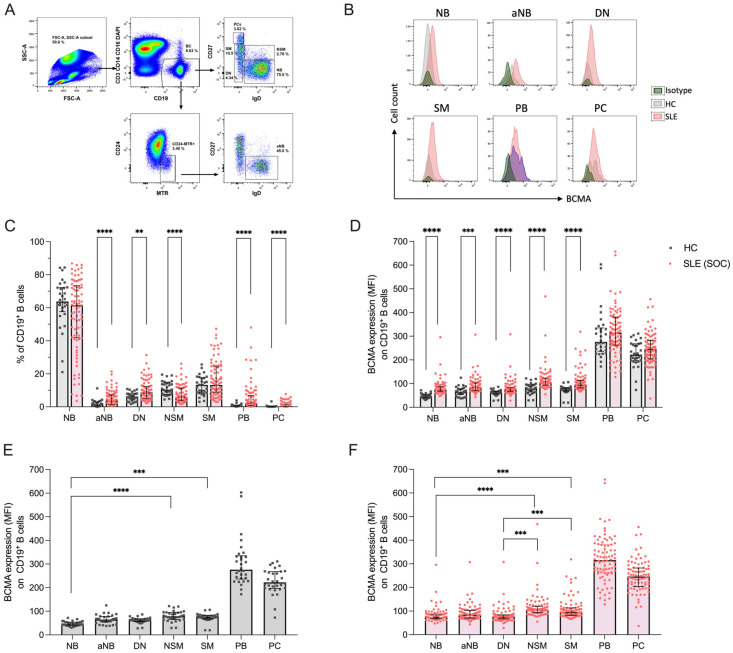
Distribution of peripheral blood B cell subsets and their surface expression of BCMA in 86 SLE patients under standard of care (SLE-SOC) and 30 HCs. (**A**) Dot plots illustrate the gating strategy of flow cytometry experiments. (**B**) Histograms show representative examples of BCMA expression in SLE, HC and isotype control (iso-ctr). (**C**) Frequencies of CD19^+^ B cell subsets, particularly IgD^+^CD27^−^ naïve B cells (NB), Mitotracker^+^IgD^+^ activated naïve B cells (aNB), IgD^−^CD27^−^ double-negative B cells (DN), IgD^+^CD27^+^ non-switched memory B cells (NSM), IgD^−^CD27^+^ switched memory B cells (SM), IgD^−^CD27^high^HLA-DR^high^ plasmablasts (PB) and IgD^−^CD27^high^HLA-DR^low^ plasma cells (PC). (**D**) BCMA expression on B cell subsets. (**E**) Comparison of BCMA expression among different B cell subsets in HCs and (**F**) in SLE-SOC. Median values with interquartile ranges are presented. Comparison between HCs and SLE-SOC was performed using the Mann–Whitney test, and comparison of BCMA expression between different B cell subsets with Kruskal–Wallis test followed by Dunn’s post hoc test for multiple comparisons. ** *p* < 0.05; *** *p* < 0.001; **** *p* < 0.0001. MFI: median fluorescence intensity.

We analyzed five CD19^+^ B cell subsets for their surface expression of BCMA, in particular IgD^+^CD27^−^ naïve (NB), Mitotracker^+^IgD^+^CD27^−^CD24^−^ activated naïve (aNB), IgD^−^CD27^−^ double-negative (DN), IgD^+^CD27^+^ non-switched memory (NSM), IgD^−^CD27^+^ switched memory (SM), as well as IgD^−^CD27^high^HLA-DR^high^ plasmablasts (PB) and IgD^−^CD27^high^HLA-DR^low^ plasma cells (PCs). The gating strategy of flow cytometry experiments is illustrated in Figure 1A, and representative BCMA staining examples are provided in Figure 1B. Compared to healthy controls, SLE patients under conventional therapy exhibited significantly higher frequencies of aNB (3.8% vs. 1.5%, *p* < 0.001), DN (8.7% vs. 6.6%, *p* = 0.003), PB (2.5% vs. 0.5%, *p* < 0.001) and PC (0.4 vs. 0.1%, *p* < 0.001) (Figure 1C). The corresponding absolute cell counts are provided in the online Supplementary Figure S1A.

When investigated for their BCMA expression, total CD19^+^ B cells expressed significantly higher levels in SLE compared to HCs (median MFI 84.7 vs. 57.9, *p* < 0.0001), while antibody-secreting IgD^−^CD27^high^ cells showed no statistical differences (Figure S1B in the online supplement). On a subset level, all investigated B cells in SLE expressed higher BCMA levels compared to HCs, particularly NB (MFI 74.4 vs. 46.0, *p* < 0.0001); aNB (MFI 85.0 vs. 65.1, *p* < 0.0001); DN (MFI 75.2 vs. 62.3, *p* < 0.0001); NSM (MFI 107.0 vs. 79.0, *p* < 0.0001); and SM (MFI 95.5 vs. 74.9, *p* < 0.0001), whereas both PB (MFI 315.5 vs. 276.0, *p* = 0.140) and PC (MFI 246.0 vs. 222.0, *p* = 0.152) displayed no significant difference (Figure 1D). We next examined whether the *surface expression* intensity of *BCMA* differed between the analyzed B cell subsets. Indeed, the highest expression levels for BCMA in HCs were found on non-switched and switched memory B cells, with significantly higher levels compared to NB (Figure 1E). Similar findings were obtained in SLE patients but with an additional significant increase in BCMA expression in both NSM and SM compared to DN B cells (Figure 1F). Collectively, these data demonstrate the upregulation of BCMA surface expression across all B cell subsets in SLE, with increased levels of memory B cells compared to naïve B cells.

### 2.2. Soluble Markers of the BAFF-APRIL System and Their Correlations

**Figure 2:**
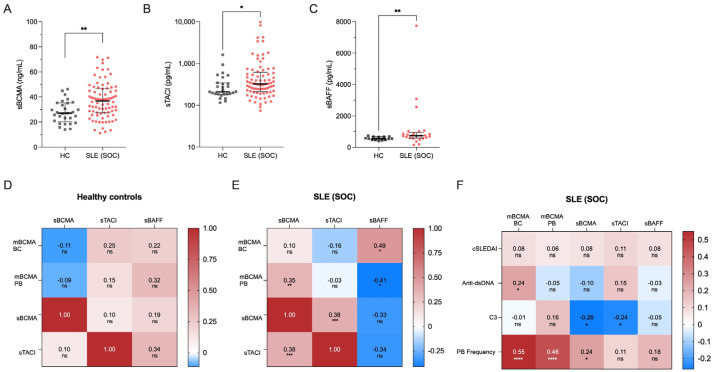
Soluble markers of the BAFF-APRIL system and their correlations in 30 healthy controls (HCs) and 86 SLE patients under standard of care (SLE-SOC). (**A**) Median values of soluble BCMA, (**B**) soluble TACI and (**C**) BAFF. (**D**) Heatmap of correlations between sBCMA, sTACI, sBAFF and BCMA expression on B cells (mBCMA BCs) in HCs, and (**E**) SLE-SOC patients. (**F**) Heatmap of correlations between BCMA expression on B cells (mBCMA BC), BCMA expression on plasmablasts (mBCMA PB), soluble BCMA, TACI and BAFF with clinical and serologic variables, particularly clinical SLEDAI-2K (cSLEDAI) scores, levels of anti-dsDNA antibodies (anti-dsDNA), complement 3 (C3) levels and plasmablast (PB) frequency. Median values with interquartile ranges are presented. Correlation analyses were performed using the Spearman ranked test, and the Spearman’s correlation coefficient (r) was calculated. ns, not significant. * *p* < 0.05; ** *p* < 0.01; *** *p* < 0.001; **** *p* < 0.0001.

We next investigated plasma concentrations of soluble BCMA, TACI and BAFF in our cohort of 86 SLE patients under conventional therapy. Compared to HCs, we found significantly higher levels of soluble BCMA (median 36.9 vs. 27.1 *p* = 0.001), TACI (median 325.7 vs. 210.0, *p* = 0.039) and BAFF (median 742.6 vs. 552.0, *p* = 0.006) (Figure 2A–C). When analyzing their interactions, we detected a moderate but significant correlation between soluble BCMA and TACI in SLE patients (r = 0.38, *p* = 0.0007) but not in HCs (Figure 2D,E). This correlation was even stronger in SLE patients treated with belimumab (r = 0.67, *p* = 0.008) (Supplementary Figure S2A). In contrast, soluble BCMA and BAFF levels were negatively correlated (r = −0.33, *p* = 0.118), and a similar trend was observed between soluble TACI and BAFF (r = −0.34, *p* = 0.100), although not reaching statistical significance.

Furthermore, we sought to determine the correlation between soluble BCMA levels and surface BCMA expression in B cells. Interestingly, sBCMA in SLE significantly correlated with surface BCMA expression on plasmablasts (r = 0.35, *p* = 0.040) but not on B cells, possibly reflecting the shedding of BCMA from the increased frequencies of PB evident in SLE (Figure 2E). In contrast, no correlation was found between sBCMA and surface expression of BCMA on B cells or plasmablasts in HCs (Figure 2D). Additionally, the surface expression of BCMA on plasmablasts in SLE negatively correlated with serum BAFF levels (r = −0.41, *p* = 0.002), whereas BCMA expression on B cells significantly correlated with serum BAFF concentrations (r = 0.49, *p* = 0.013) (Figure 2E).

Collectively, all examined soluble components of the BAFF-APRIL system were upregulated in SLE with moderate correlations between soluble BCMA and TACI and between soluble BCMA and surface BCMA on PB and soluble BAFF on B cells. Remarkably, no significant correlations were detected in healthy controls, highlighting the chronic upregulated humoral activity and disturbed immunological homeostasis in SLE.

### 2.3. Correlations of BCMA with Clinical and Serologic Variables and Plasmablast Frequency

We finally evaluated the complex interactions of soluble BCMA and BCMA expression on B cells and plasmablasts, with markers of clinical and serologic disease activity, as well as frequencies of circulating plasmablasts. Interestingly, no correlations were found between soluble BCMA or surface BCMA expression and clinical disease activity in SLE, as measured by clinical domains of the SLEDAI-2K (Figure 2E). In contrast, we found significant correlations between BCMA expression on B cells and serum anti-dsDNA antibody levels (r = 0.24, *p* = 0.031) and between soluble BCMA (r = −0.26, *p* = 0.017) and complement consumption for C3 (Figure 2F). More importantly, both soluble BCMA (r = 0.24, *p* = 0.029) and surface BCMA on B cells (r = 0.55, *p* < 0.0001) and plasmablasts (r = 0.46, *p* < 0.0001) strongly correlated with the frequency of circulating plasmablasts among CD19^+^ B cells, identifying these markers as surrogates for humoral activity in SLE. These findings were confirmed by using absolute cell counts (Figure S2C,D) and in patients undergoing belimumab treatment (Figure S2B).

### 2.4. Impact of Belimumab Treatment on the BAFF/APRIL System

Previous studies have demonstrated that the BAFF-targeting monoclonal antibody belimumab impacts the BAFF/APRIL system by inhibiting the binding of soluble BAFF to its receptors (BAFF receptor, TACI and BCMA) expressed on B cells [40]. Therefore, we aimed to evaluate BCMA expression on B cell subsets in an independent cohort of 14 SLE patients under stable treatment with intravenous belimumab (SLE-BEL) in comparison to SLE patients receiving conventional therapy (SLE-SOC). The clinical characteristics of the belimumab-treated patients are provided in Table S1.

**Figure 3:**
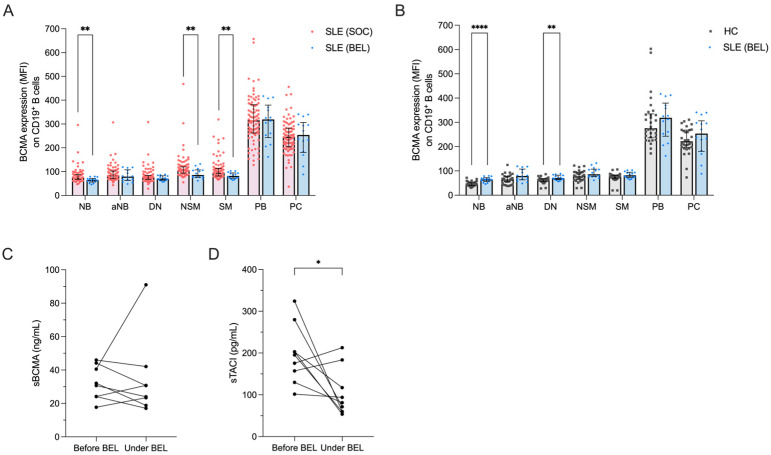
Effects of belimumab treatment on BCMA expression on B cell subsets. (**A**) BCMA expression levels on different B cell subsets in 86 SLE patients under standard of care (SLE-SOC) compared to 14 SLE patients under belimumab treatment (SLE-BEL). (**B**) BCMA expression on different B cell subsets in SLE-BEL patients compared to 30 HCs. (**C**) Levels of soluble BCMA (sBCMA) and (**D**) soluble TACI (sTACI) in a cohort of 9 SLE patients before and at a median of 8 months after initiating belimumab treatment. Median values with interquartile ranges are presented. The Mann–Whitney U test was performed to compare SLE-SOC with SLE-BEL and HCs. The Wilcoxon signed-rank test was used to compare sBCMA and sTACI before and during belimumab treatment. * *p* < 0.05; ** *p* < 0.01; **** *p* < 0.0001.

Consistent with previous findings, we noted significant reductions in the frequencies of naïve B cells (21.3% vs. 61.5%, *p* < 0.0001) and activated naïve B cells (1.3% vs. 3.8%, *p* = 0.006), along with increased frequencies of non-switched (11.0% vs. 6.1%, *p* = 0.015) and switched memory B cells (44.6% vs. 13.2%, *p* < 0.0001) in belimumab-treated compared to conventionally treated SLE patients (Figure S3A) [41,42]. Surface BCMA expression levels were significantly lower in belimumab- vs. conventionally treated patients on naïve B cells (74.4 vs. 65.1, *p* = 0.001), non-switched (107.0 vs. 86.1, *p* = 0.008) and switched memory B cells (95.5 vs. 82.0, *p* = 0.005) (Figure 3A). This resulted in a normalization of BCMA expression on memory B cell subsets in belimumab-treated SLE patients compared to HCs, but BCMA expression remained elevated on naïve and double-negative memory B cells (Figure 3B).

Finally, we prospectively followed a cohort of nine SLE patients after initiation of belimumab treatment to assess changes in their soluble BCMA and TACI levels. The disease characteristics of these patients are provided in Table S2. After a median follow-up of 8 months (range 5–39 months), circulating plasmablasts decreased significantly (from 19.7% to 4.4% among CD19^+^ B cells, *p* = 0.004) (Figure S3B). Plasma concentrations of soluble BCMA did not show significant changes during this period (median 31.4 vs. 27.4 ng/mL, *p* = 0.547) (Figure 3C), whereas soluble TACI significantly decreased (median 195.3 vs. 81.2 pg/mL, *p* = 0.039) (Figure 3D).

## 3. Discussion

Given the central role of BCMA and its ligands BAFF and APRIL in the dysregulated B cell homeostasis observed in SLE, we investigated the complex interplay between BCMA expression on peripheral blood B cells subsets, soluble BCMA plasma concentrations and clinical disease variables as well as peripheral blood plasmablast expansion, a hallmark of the disease. Our data reveal significantly increased BCMA expression levels on all investigated B cell subsets in SLE, which strongly correlated with serum anti-dsDNA antibody levels and the frequency of circulating plasmablasts, with the latter also correlating with soluble BCMA, identifying these markers as surrogates for disease activity as well as biomarkers for humoral activity in SLE. More importantly, we found higher BCMA expression levels on memory compared to naïve B cells, highlighting BCMA as an attractive therapeutic target for preferential depletion of memory B cell subsets along with plasmablasts and plasma cells constitutively expressing BCMA.

In contrast to the BAFF receptor, BCMA is upregulated on activated B cells, reflected by increased BCMA expression along with other B cell activation markers, such as CD86 [26] and CD38 [29], and activation of B cells by CpG/IL-2/IL-15 induced BCMA expression in vitro [31]. In line with this notion, we found significantly increased surface BCMA levels on all investigated B cell subsets, but not plasmablasts, in SLE patients. The hierarchy of BCMA expression from lowest to highest was naïve B cells, double-negative memory B cells, activated naïve B cells, switched memory B cells, non-switched memory B cells, plasma cells and plasmablasts. Our findings align with those of Kim et al. [26] and Álvarez Gómez et al. [37] but differ from the earlier studies by Salazar-Camarena et al. [36,39]. The latter used the frequency of BCMA-positive B cells for correlation analyses rather than the expression density based on the mean fluorescent intensity (MFI) values on B cells. These studies reported lower frequencies of BCMA-positive B cells in SLE patients compared to healthy controls, along with a negative correlation with the Mexican (MEX)-SLEDAI score. Our study differs from the latter two publications in other clinical features, in particular overall lower disease activity and the absence of treatment-naïve patients, which might explain the different results. Additionally, Kim et al. found elevated BCMA expression on memory B cells and especially on plasmablasts and plasma cells, whereas BCMA expression on naïve B cells was relatively low [26]. Álvarez Gómez et al. reported increased BCMA expression on resting naïve B cells and switched memory B cells but not on DN and activated naïve B cells [37]. This could be due to differences in the definition of these subgroups, with their study defining DN in subgroups of IgD^−^ CD27^−^ CXCR5^+^ CD11c^−^ (DN1) and IgD^−^ CD27^−^ CXCR5^−^ CD11c^+^ (DN2) cells and activated naïve B cells as IgD^+^ CD27^−^ CXCR5^−^ CD11c^+^.

Collectively, our data confirm and extend previous findings and demonstrate that increased BCMA expression on B cells is a common and reproducible feature of SLE, with memory B cells displaying higher expression levels compared to naïve B cell subsets. This notion could be relevant for BCMA-targeted therapies, in which naïve B-cell subsets might be spared from depletion. BCMA is already an established target in the treatment of multiple myeloma, using either bispecific antibody constructs, antibody–drug conjugates or chimeric antigen receptor (CAR)-modified T cell therapies [43], and could represent an attractive novel therapeutic target in SLE for depleting plasma cells together with memory B cells. To this end, our recent data on the CD3-BCMA bispecific antibody teclistamab show a complete depletion of plasma cells in the bone marrow, accompanied by a preferential depletion of memory over naïve B cell subsets among the few remaining B cells after treatment, resulting in complete remission “off-therapy” [10]. However, a longer follow-up period and data from a larger patient population are required to determine the duration of response and evaluate long-term side effects.

Membrane-bound BCMA can undergo γ-secretase-mediated shedding from the cell surface, leading to the circulation of soluble BCMA and reduced activation of surface BCMA by BAFF and APRIL [34]. Furthermore, BAFF and APRIL are ligands of BCMA and TACI, with BAFF also binding exclusively to the BAFF receptor, which is essential for B cell proliferation and survival [44]. In our cohort of patients, plasma concentrations of BCMA, BAFF and TACI were significantly increased compared to healthy controls, consistent with previous reports in the literature [34,35,36,45,46]. Additionally, we observed a strong correlation between sBCMA and sTACI, but not sBAFF, in SLE patients. Soluble TACI binds to BAFF, leading to its inhibition [45], potentially explaining the negative correlation between BAFF and sTACI. TACI shedding is mediated by ADAM10 independently of BAFF [47].

In vitro, concentrations of soluble BCMA shedded by γ-secretase correlated with membrane BCMA levels in B cells [34]. We, therefore, expected to detect a correlation between sBCMA and BCMA expressed in B cells, but this was not evident. Instead, sBCMA strongly correlated with the BCMA expression on plasmablasts, probably because they display the highest BCMA expression levels and are increased in SLE. More strikingly, sBCMA strongly correlated with the frequency of circulating plasmablasts. Thus, sBCMA levels may serve as a useful surrogate for humoral activity in SLE, similar to findings in multiple myeloma, where sBCMA levels are elevated and correlate with the proportion of myeloma cells in the bone marrow [48]. This finding could have important implications for the management of SLE in clinical practice. Increased circulating plasmablast numbers are well recognized as a biomarker for disease activity [49] and for stratifying responses to B-cell-targeted therapies [50]. However, this analysis requires a flow cytometric examination of freshly isolated blood, which is not generally available in clinical routine and could be replaced by the measurement of soluble BCMA in serum. To what extent increased BCMA levels could be used for diagnostics remains to be determined.

BCMA expression on B cells is linked to their activation and maturation, particularly on CD27^+^ memory B cells and antibody-secreting cells [31]. Consequently, increased BCMA expression on B cells may result in elevated autoantibody production. Indeed, we observed a moderate correlation between BCMA expression on B cells and serum anti-dsDNA levels. In addition, negative correlations were found between serum C3 levels and both sBCMA and sTACI, suggesting that high BCMA and TACI expression promotes autoantibody production and immune complex formation. Consistent with these findings, Laurent et al. reported a negative correlation between sBCMA and C3 levels in SLE [34], collectively identifying membrane-bound BCMA on B cells as a biomarker for disease activity.

There is ample evidence that the monoclonal antibody belimumab, which targets BAFF, can at least partially restore the impaired B-cell homeostasis in SLE by reducing B-cell activation and inhibiting the differentiation of autoreactive antibody-secreting cells through reduced binding of BAFF to its receptors BAFF receptor, TACI and BCMA [41,42,51]. Indeed, our cohort of belimumab-treated patients had lower BCMA expression on naïve and memory B cells than conventionally treated patients, with BCMA expression on non-switched and switched memory B cells reaching similar levels to healthy controls. This suggests that these B cell subgroups are preferentially inhibited by belimumab and are more dependent on BAFF signaling than plasma cells, which maintained BCMA expression levels under belimumab, presumably due to a stronger dependency on APRIL, which also binds BCMA and TACI. There was a significant correlation between soluble BAFF and BCMA expression on B cells in conventionally treated patients but not in belimumab-treated patients, suggesting that BAFF inhibition is the most important factor in downregulating BCMA expression on B cells. Our findings align with previous studies, which reported a significant decrease in BCMA MFI on total B cells after three months of belimumab treatment [40]. However, they did not analyze membrane-bound BCMA at the B cell subset level. Furthermore, intraindividual reductions in sTACI levels were observed during belimumab treatment, consistent with Piantoni et al. [52]. This indicates that belimumab reduces TACI expression on B cell membranes, as shown by Hirano et al. [40]. Piantoni et al. also reported intraindividual decreases in sBCMA during belimumab treatment [52], providing more reliable data than in our study because they included more treated patients with defined time points [53]. Taken together, these results support the notion that neutralization of BAFF by belimumab inhibits the entire BAFF-APRIL system and downregulates its receptors in the long term, leading to reduced plasmablast frequencies and clinical efficacy.

The strength of our study is the evaluation of BCMA surface expression at the B cell subset level in a large cohort of SLE patients, combined with the analysis of the soluble ligands of BCMA. However, our results may be confounded by the heterogeneity of clinical manifestations and underlying treatments among the investigated patients. Notably, the cohort of belimumab-treated patients is relatively small, and most data were obtained cross-sectionally, with a lack of longitudinal analysis of BCMA expression on B cells. This must be taken into account when interpreting the data, as confounding factors cannot be ruled out. Nevertheless, our study provides a detailed analysis of BCMA-expressing B cells and their correlations with clinical variables and is probably one of the most comprehensive analyses of the BAFF/APRIL system in SLE to date. Future investigations could benefit from the inclusion of transcriptomic or metabolomic data and functional analyses of BCMA-expressing B cells to complement our phenotypic evaluations.

In conclusion, we identified increased BCMA expression on B cell subsets as a prominent and reproducible feature of SLE. BCMA expression on B cells strongly correlates with the frequency of circulating plasmablasts and serum anti-dsDNA antibody titers, and it is downregulated with belimumab treatment. Soluble BCMA correlates with the level of BCMA expression on plasmablasts and their frequency in peripheral blood, identifying these markers as surrogates for clinical and humoral activity that could be utilized for monitoring SLE patients in clinical routine. Furthermore, we found that BCMA expression is significantly higher on memory B cell subsets compared to their naïve counterparts, positioning BCMA as a potential therapeutic target for depleting plasma cells and their memory B cell precursors using monoclonal or bispecific antibodies, as well as BCMA-directed CAR-T cell therapies.

## 4. Materials and Methods

### 4.1. Patient and Control Blood Samples

We recruited 100 patients with SLE according to the 2019 EULAR/ACR classification criteria [54] from the Department of Rheumatology and Clinical Immunology at Charité-Universitätsmedizin Berlin between April and November 2016. As controls, we included 30 age- and gender-matched healthy individuals. Clinical and serologic manifestations, including the SLEDAI-2K [55], and immunosuppressive medication regimes were recorded by the treating physician. Patients were subdivided according to their treatment into groups of those with (*n* = 14) or without (*n* = 86) belimumab. Patients in the belimumab group were under intravenous belimumab therapy for a median duration of 9 months (range 5–39 months), and their clinical characteristics are provided in Table S1. All samples were collected 4 weeks after the previous belimumab infusion. From 9 out of 14 patients with belimumab treatment, plasma was collected for the analysis of soluble BCMA and TACI.

### 4.2. Cell Isolation and Flow Cytometry

For flow cytometry, 5 mL of freshly obtained heparinized blood was mixed with 50 mL of Red Blood Cell Lysis Buffer (BD Biosciences, San Jose, CA, USA) in Falcon tubes for 10 min to eliminate red blood cells. After centrifugation, samples were washed with phosphate-buffered saline containing bovine serum albumin and centrifuged again to generate peripheral blood mononuclear cells (PBMCs). They were incubated with MitoTracker Deep Red (MTR, Thermo Fisher Scientific, Waltham, MA, USA) at a concentration of 1:1,000,000 at 37 °C for 10 min, washed and centrifuged. Subsequently, cells were stained in 100 µL PBS for 20 min at 4 °C with the antibodies indicated in Supplementary Table S3. Fluorescence-minus-one (FMO) controls were used for compensation and quality control. DAPI was added to stain dead cells, and samples were acquired on a FACSCanto II (BD Biosciences, San Jose, CA, USA). FACS analysis was performed following the guidelines for the use of flow cytometry and cell sorting in immunological studies [55]. Cytometry data were analyzed using FlowJo v10.6.2 (FlowJo, LLC, Ashland, OR, USA).

### 4.3. ELISA

Heparinized blood samples were centrifuged for 10 min to obtain plasma from each participant, which was frozen at −20 °C for subsequent analysis. ELISA was performed to analyze plasma levels of BAFF (BAFF/BLyS Quantikine), TACI (DuoSet) and BCMA (DuoSet), all R&D Systems, NE Minneapolis, MN, USA, according to the manufacturer’s protocols. SoftMax Pro V.6.5 was used to analyze ELISA results.

### 4.4. Statistical Analysis

Statistical analysis was conducted using SPSS Statistics version 29.0, while figures were generated with GraphPad Prism version 9.0. To compare data between different cohorts, we utilized the Mann–Whitney U test. Within the same cohort, comparisons of B cell subsets were performed using the Kruskal–Wallis test followed by Dunn’s post hoc test for multiple comparisons. Intraindividual changes under belimumab treatment were assessed by applying the Wilcoxon signed-rank test. Spearman’s rank correlation test was employed for correlation analyses. Statistical significance was defined as a *p* value of less than 0.05. In the figures, statistical significance was represented by the following symbols: ns or no symbol means *p* > 0.05, * means *p* < 0.05, ** means *p* < 0.01, *** means *p* < 0.001 and **** means *p* < 0.0001. In the results section, comparison values presented in brackets always represent medians.

## Figures and Tables

**Table 1:** Demographics and clinical characteristics of investigated SLE patients and controls.

Characteristics	SLE Patients (*n* = 100)	Healthy Controls (*n* = 30)	*p*-Value
Age, median (range)	38.5 (19–80)	28.7 (22–59)	0.11
Gender, female, *n* (%)	90 (90)	26 (86.7)	0.61
Ethnicity, *n* (%)			
Caucasian	90 (90)	26 (86.6)	0.61
Asian	1 (1)	2 (6.7)	0.07
African	6 (6)	2 (6.7)	0.89
Latin American	3 (3)	0 (0)	0.34
Disease duration, median years, (range)	6.5 (6–40)		
Disease activity			
SLEDAI-2K, median (range)	4 (0–26)		
Clinically active, *n* (%)	46 (46)		
DORIS remission, *n* (%)	54 (54)		
Active clinical manifestations at time of presentation, *n* (%)			
Musculoskeletal	33 (33)		
Mucocutaneous	18 (18)		
Polyserositis	3 (3)		
Nephritis	5 (5)		
CNS	2 (2)		
Cytopenia	43 (43)		
Serology			
Anti-dsDNA-positive, *n* (%)	71 (71)		
Serum anti-dsDNA levels (IU/mL), median	45 (4–200)		
(range)			
C3-deficiency, *n* (%)	71 (71)		
Serum C3 levels (mg/L), median (range)	820 (330–1330)		
Medication, *n* (%)			
Prednisolone	75 (75)		
Prednisolone dosage (mg/d),	5.0		
median			
Prednisolone ≥ 7.5 mg/d	30 (30)		
Hydroxychloroquine	78 (78)		
Methotrexate	11 (11)		
Azathioprine	34 (34)		
Mycophenolate mofetil	14 (14)		
Calcineurin inhibitors	5 (5)		
Belimumab	14 (14)		

Abbreviations: SLEDAI-2K, Systemic Lupus Erythematosus Disease Activity Index 2000. Statistical analysis of age differences was conducted using the Mann–Whitney test, and sex differences were analyzed with the chi-square test.

## Data Availability

The data from this study are available from the corresponding author upon request (due to legal reasons).

## Supplementary Materials

The following supporting information can be downloaded at: https://www.mdpi.com/article/10.3390/ijms251910845/s1.
