# Supplementary material for: B-Cell Maturation Antigen (BCMA) as a Biomarker and Potential Treatment Target in Systemic Lupus Erythematosus
Source: Int J Mol Sci. 2024 Oct 9;25(19):10845. doi: 10.3390/ijms251910845 (PMC11476889; doi:10.3390/ijms251910845)
Supplement: Supplementary file 1 [file ijms-25-10845-s001.zip › ijms-3208631-supplementary.pdf]

Supplementary Material

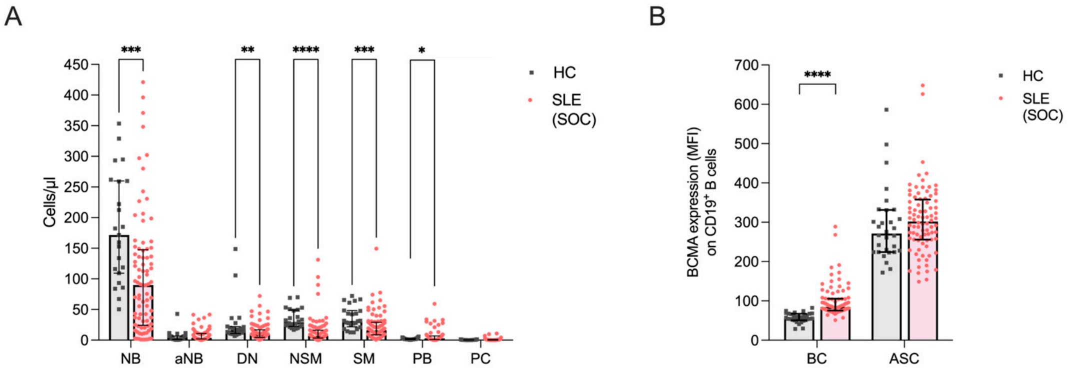

Figure S1. Absolute cell counts of different B cell subsets and BCMA expression on total B cells and antibody secreting sells. Comparison between 30 healthy controls (HC) and 86 SLE patients undergoing standard of care immunosuppression (SLE SOC) using Mann-Whitney U test. **(A)** comparison between HC and SLE SOC patients of absolute numbers of different B cell subsets in trucount measurements. **(B)** comparison between HC and SLE SOC patients of BCMA expression on total B cells and antibody secreting cells. Black lines represent the interquartile range with median values. B cell subsets include naive B cells (NB), activated naive B cells (aNB), double-negative memory B cells (DN), non-switched memory B cells (NSM), switched memory B cells (SM), plasmablasts (PB), and plasma cells (PC). * $p < 0.05$; ** $p < 0.01$; *** $p < 0.001$; **** $p < 0.0001$; MFI: median fluorescence intensity.

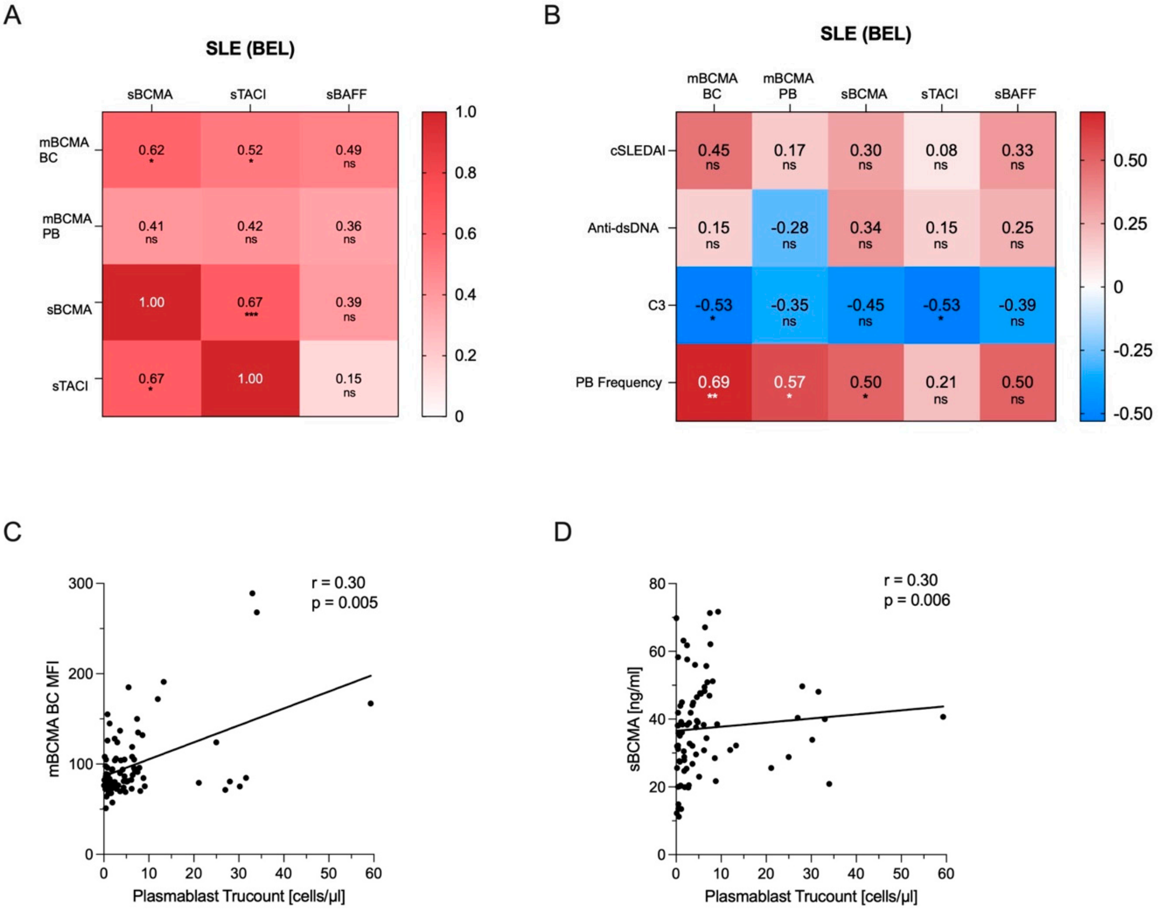

Figure S2. Plasma levels of soluble BCMA (sBCMA), soluble TACI (sTACI), soluble BAFF (sBAFF) and BCMA expression (mBCMA) on total B cells (BC) and plasmablasts (PB). Correlation analyses for 14 SLE patients undergoing belimumab treatment (BEL) (Figure S2A-B) and 86 SLE patients undergoing standard of care immunosuppressive treatment (SOC)

(Figure S2C-D) with Spearman ranked test and calculation of Spearman's rank correlation coefficient (*r*) and p-values (*p*). **(A)** correlation matrix heat map from SLE BEL patients representing r-values and levels of significance for sBCMA, sTACI, sBAFF, BCMA expression on total B cells (mBCMA BC) and BCMA expression on plasmablasts (mBCMA PB). **(B)** correlation matrix heat map from SLE BEL patients representing r-values and levels of significance for BCMA expression on total B cells (mBCMA BC), BCMA expression on plasmablasts (mBCMA PB), sBCMA, sTACI and sBAFF, correlated with clinical markers clinical SLEDAI-2K (cSLEDAI), levels of anti-dsDNA-antibodies (anti-dsDNA), complement 3 (C3) levels, and plasmablast (PB) frequency. **(C)** correlation of plasmablast trucount with soluble BCMA in SLE SOC patients. **(D)** correlation of plasmablast trucount with BCMA expression on total B cells in SLE SOC patients. * $p < 0.05$; ** $p < 0.01$; *** $p < 0.001$; **** $p < 0.0001$.

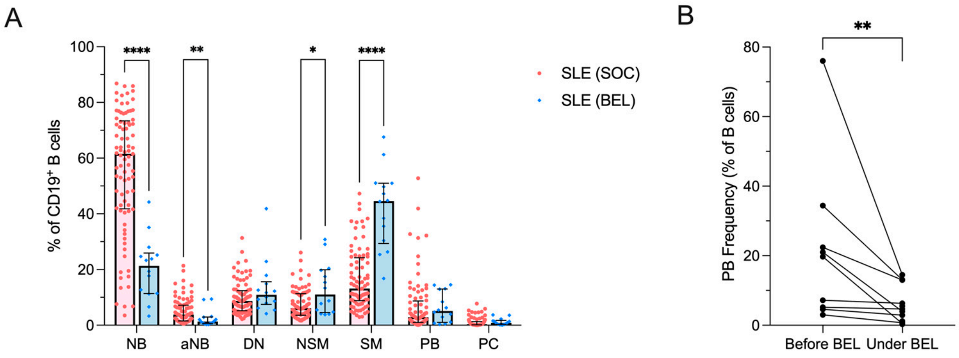

Figure S3. Intraindividual Effects of Belimumab Treatment and Effects of Belimumab Treatment on the Distribution of B Cell Subsets. Comparison between 86 SLE patients undergoing standard-of-care immunosuppression (SOC), and 14 on belimumab treatment (BEL), using Mann-Whitney-U-test. Black lines represent the interquartile range with median values. Comparison of values from before and during belimumab treatment (BEL) in 9 SLE patients, at a median of 8 months after initiating BEL, using Wilcoxon signed-rank test. Frequencies of displayed B cell subsets include naive B cells (NB), activated naive B cells (aNB), double-negative memory B cells (DN), non-switched memory B cells (NSM), switched memory B cells (SM), plasmablasts (PB) and plasma cells (PC). p-values (*p*). **(A)** distribution of different B cell subsets among total B cells between SLE patients under SOC and under BEL treatment. **(B)** plasmablast frequency Black lines represent the interquartile range with median values. B cell subsets include naive B cells (NB), activated naive B cells (aNB), double-negative memory B cells (DN), non-switched memory B cells (NSM), switched memory B cells (SM), plasmablasts (PB), and plasma cells (PC). * $p < 0.05$; ** $p < 0.01$; *** $p < 0.001$; **** $p < 0.0001$.

Table S1. Comparison of Patients with Standard of Care Immunosuppression versus Patients Receiving Belimumab Treatment.

Characteristics	Standard of care (n=86)	Belimumab (n=14)	P-Value
Age, median (range)	37.0 (19–80)	41.3 (24–63)	0.26
Gender, n (%) Female	78 (91)	12 (86)	0.56
Ethnicity, n (%)			
Caucasian	80 (93)	10 (72)	0.01
Asian	0 (0)	1 (7)	0.01
African	4 (5)	2 (14)	0.09
Latin American	2 (2)	1 (7)	0.33
Disease duration, median years (range)	6.5 (0–40)	8.6 (1–27)	0.66
SLEDAI-2K, median (range)	4 (0–26)	7 (0–10)	0.13
Clinically active, n (%)	37 (43)	9 (64)	
DORIS remission, n (%)	23 (27)	3 (21)	
Clinical manifestations at time of presentation, n (%)			

Musculoskeletal	27 (31)	6 (43)	
Mucocutaneous	14 (16)	4 (29)	
Polyserositis	2 (2)	1 (7)	
Nephritis	5 (6)	0 (0)	
CNS	2 (2)	0 (0)	
Cytopenia	39 (45)	4 (29)	
Serology			
Anti-dsDNA positive, n (%)	60 (70)	11 (79)	
Serum anti-dsDNA levels (IU/mL), median (range)	47 (4–200)	45 (4–200)	0.81
C3-deficiency, n (%)	60 (70)	11 (79)	
Serum C3 levels (mg/L), median (range)	825 (330–1330)	745 (480–1050)	0.12
Medication, n (%)			
Prednisolone	61 (71)	14 (100)	
Prednisolone dosage (mg/d), median	5.0	7.25	0.03
Prednisolone ≥ 7.5 mg/d	23 (27)	7 (50)	
Hydroxychloroquine	71 (83)	7 (50)	
Methotrexate	8 (9)	3 (21)	
Azathioprine	28 (33)	6 (43)	
Mycophenolate mofetil	14 (16)	0 (0)	
Calcineurin inhibitors	4 (5)	1 (7)	
Belimumab, median treatment duration in months (range)	n.a.	9.0 (5–39)	

Abbreviations: SLEDAI-2K, Systemic Lupus Erythematosus Disease Activity-Index 2000. Statistical analysis of age differences was conducted using the Mann-Whitney test, and sex differences were analyzed with the chi-square test.

Table S2. Belimumab-Treated Patients Included in Intraindividual Analyses.

Characteristics	n=9
Age, median (range)	41.6 (24–63)
Gender, n (%) Female	8 (89)
Ethnicity, n (%)	
Caucasian	5 (56)
Asian	1 (11)
African	2 (22)
Latin American	1 (11)
Disease duration [years], mean (range)	3.4 (1–27)
SLEDAI-2K, median (range)	8 (4-10)
Clinically active, n (%)	7 (78)
DORIS remission, n (%)	2 (22)
Clinical manifestations at time of presentation, n (%)	
Musculoskeletal	6 (67)
Mucocutaneous	7 (78)
Polyserositis	0 (0)
Nephritis	0 (0)
CNS	0 (0)
Cytopenia	7 (78)
Serology	
Anti-dsDNA positive, n (%)	7 (78)
Serum anti-dsDNA levels (IU/mL), median (range)	53 (5–159)
C3-deficiency, n (%)	7 (78)
Serum C3 levels (mg/L),	750 (480–960)

median (range)	
Medication, n (%)	
Prednisolone	9 (100)
Prednisolone dosage (mg/d), median	10.0
Prednisolone ≥ 7.5 mg/d	7 (78)
Hydroxychloroquine	5 (56)
Methotrexate	1 (11)
Azathioprine	5 (56)
Mycophenolate mofetil	0 (0)
Calcineurin inhibitors	1 (11)
Belimumab, median treatment duration in months (range)	8.0 (5-26)

Abbreviations: SLEDAI-2K, Systemic Lupus Erythematosus Disease Activity-Index 2000.

Table S3. FACS Panels and Applied Fluorescent Dyes.

Panel 1: Antigens and dyes (concentration) (clone and manufacturer)	Panel 2: Antigens and dyes (concentration) (clone and manufacturer)
IgD FITC (1:20) (Clone IA 6-2, BioLegend, USA)	IgD FITC (1:20) (Clone IA 6-2, BioLegend, USA)
CD269/BCMA PE (1:20) (Clone 19F2, BioLegend, USA)	Isotype control PE (1:20) (Clone QA16A12, BioLegend, USA)
CD24 PerCP (1:20) (Clone ML5, BioLegend, USA)	CD24 PerCP (1:20) (Clone ML5, BioLegend, USA)
CD19 PE/Cy7 (1:20) (Clone SJ25c1, BioLegend, USA)	CD19 PE/Cy7 (1:20) (Clone SJ25c1, BioLegend, USA)
Mito Tracker Deep Red (1:1000000) (Thermo Fisher, USA)	
CD27 APC/Cy7 (1:20) (Clone O323, BioLegend, USA)	CD27 APC/Cy7 (1:20) (Clone O323, BioLegend, USA)
HLA-DR Brilliant Violet 510 (1:20) (Clone L243, BioLegend, USA)	HLA-DR Brilliant Violet 510 (1:20) (Clone L243, BioLegend, USA)
CD3 Pacific Blue (1:20) (Clone UCHT1, BD Biosciences, USA)	CD3 Pacific Blue (1:20) (Clone UCHT1, BD Biosciences, USA)
CD14 Pacific Blue (1:20) (Clone MϕP9, BD Biosciences, USA)	CD14 Pacific Blue (1:20) (Clone MϕP9, BD Biosciences, USA)
CD16 Pacific Blue (1:20) (Clone MOPC-21, BioLegend, USA)	CD16 Pacific Blue (1:20) (Clone MOPC-21, BioLegend, USA)
DAPI Pacific Blue (1:20)	DAPI Pacific Blue (1:20)
